# A qualitative study on the challenges of clinical leadership in an HIV care system: insights from healthcare providers in Eldoret, Kenya

**DOI:** 10.3389/frhs.2025.1404902

**Published:** 2025-05-14

**Authors:** Felishana Cherop, Michael Korir, Vincent Bagire, Andrew Kimwolo, Violet Naanyu, Juddy Wachira

**Affiliations:** ^1^ Department of Management Science & Entrepreneurship, School of Business and Economics Moi University, Eldoret, Kenya; ^2^ Department of Business Administration, Makerere University Business School (MUBS), Kampala, Uganda; ^3^ Department of Sociology and Anthropology, School of Arts & Social Sciences, Moi University, Eldoret, Kenya; ^4^ Department of Mental Health and Behavioral Sciences, School of Medicine, Moi University, Eldoret, Kenya

**Keywords:** clinical leadership, challenges, HIV care, healthcare providers, health system

## Abstract

**Introduction:**

The provision of quality services to patients in healthcare facilities requires effective clinical leaders who will transcend their technical expertise and coordinate and direct patient care through clinical leadership roles. Clinical leadership refers to using clinical experience to provide direction, inspire and promote values and vision, and promote quality clinical care. However, there is a limited understanding of the challenges faced by clinical leaders within HIV care systems in Kenya. This study explored the views of healthcare providers on clinical leadership challenges in HIV care highlighting the sources and consequences.

**Methods:**

We conducted an exploratory qualitative study between December 2019 to May 2020 marked by COVID-19 involving (*n* = 22) healthcare providers who were purposively sampled to participate in in-depth interviews in the AMPATH-MTRH HIV facility in Eldoret, Kenya. Ethics approval was granted, and participants consented to participation and audio-recorded interviews. All data that were collected from participants were de-identified and kept in a confidential format to protect participant anonymity. A thematic analysis approach was used to analyze data and Nvivo v.12 software was used for data management.

**Results:**

Participants identified three broad themes that described clinical leader challenges in an HIV facility including (1) supply-side challenges, a shortage of resources, staff welfare, and team dynamics; (2) demand-side challenges, unmet patient expectations, lack of appreciation by the patients, lack of additional gains and incentives, financial constraints, and stigma; (3) health system challenges, rigid health system structure, lack of management support, unavailable services in the facility. These challenges negatively impacted healthcare providers' performance including clinical leaders', compromised patient care, and created inefficiencies in the HIV care system.

**Conclusion:**

The results provided important insights from the perspectives of healthcare providers. They show that in HIV care systems, clinical leaders are faced with diverse challenges that emerge from the supply, demand, and health system sides that affect patient care and system performance. Healthcare system leaders can strengthen management support systems and leadership training for clinical leaders to improve HIV care provision as well as provide career growth opportunities for clinical leaders to maximize their expertise in improving HIV care and system performance.

## 1 Introduction

In healthcare organizations, clinical leaders play important roles in providing leadership to enhance patient care and health system performance (1–3). However, clinical leadership has received limited attention in low- and medium-income countries (LMICs) due to the cultures, norms, and structures that constrain leadership practice (4). Clinical leadership is described as the use of clinical experience to provide direction, inspire and promote values and vision, and promote quality clinical care to ensure the needs of the patients are prioritized within the organization's aims and delivery (5). Similarly, it is defined as a distinct and emergent phenomenon resulting from the dynamic interactions within a healthcare system that requires high clinical competency to produce optimal care by having the purpose and qualities of delivering change in the quality of direct patient care (6). The definitions highlight both the capacity and experience of clinical leaders. In the context of this study, we refer to clinical leadership as the role of clinicians with competency and experience to provide care and perform leadership and management roles to see that services to HIV patients are effective and satisfactory and meet the goals of the HIV care system. A recent study established that clinical leaders in HIV primary care facilities provide strategic direction, ensure resource allocation, interconnect health systems, provide supervisory oversight roles, and oversee research to enhance patient care, which requires strengthening their capacity to maximize clinicians' contribution to improve HIV care and enhance responsive health systems (7). It is important to include clinicians at all levels of the organization's leadership from the chief executive officer (CEO) to managing a small clinic to ensure the clinical environment operates in a safe, good-quality, and effective way to meet the needs of the patients (5). However, ineffective clinical leadership at the frontline can have a stark consequence on the quality and outcomes of care (8). Clinical leadership has been perceived to promote integrative and proactive care through the facilitation of interdisciplinary collaboration (9). In Kenya, the use of distributed leadership to examine clinical leadership was important in analyzing middle-level leadership. Distributed leadership refers to a shared approach to leadership, where leadership responsibilities are distributed across multiple actors within a healthcare system. For example, in Kenyan county hospitals, this model highlights the role of middle-level clinical leaders, such as doctors and nurses, in influencing their peers, coordinating care, and adapting leadership practices to local contexts (4). In addition, during the COVID-19 pandemic, the establishment of a hospital leadership structure was effective and efficient in determining preparedness and response strategies for the COVID-19 pandemic in Kenya (10), underscoring the importance of leadership at all levels of management including clinical leadership at the operational level to respond to public health pandemics. However, there is insufficient data on whether clinical leadership challenges in an HIV care system could affect HIV patient care and system improvement. In an aged facility, clinical nurse leaders who had more than 10 years of experience and older than 50 years had higher levels of self-confidence in clinical leadership roles (11) indicating an understanding of the clinical environment. However, there is scarce literature on the clinical leadership contribution to HIV care particularly in Kenya.

Although clinical leaders play important roles in healthcare organizations, there are leadership challenges associated with their roles. For example, in Kenyan hospitals, clinical leadership is challenged by contextual factors such as cultures, norms, and structures that influence how clinical leadership is practiced (4). This would inform the structure of clinical leadership and the experience of the clinical leaders such as doctors, clinical officers, and nurses, who take on leadership roles within hospital departments and in navigating the dynamics within the hospital contexts. In Nigeria, participants perceived the involvement of clinicians in organizational leadership roles as having little to no benefit for patient care or their professional success. The perception contributed to a lack of motivation for clinicians to pursue leadership roles, which in turn affected healthcare delivery. In addition, the lack of leadership training in medical curricula left clinicians unprepared for management roles, further limiting their influence on healthcare delivery (12). This would then create gaps in day-to-day healthcare service delivery. In the context of HIV, the challenges that impede leadership in collaborative governance include divergence of representation and participation in terms of obtaining consensus toward a common purpose, managing different personalities, time management to collaborative process, and leadership incapacities, all of these create unique challenges to the leaders (13). In addition, while effective clinical leadership principles integrate relational, personal, clinical, and evidence-based practice and optimal care management (14), however, its effectiveness may vary across contexts due to factors such as staffing, resources, and patient–provider dynamics. In Nigeria, the clinical leadership in labor wards does not necessarily work differently from those in other countries, but the clinical leadership challenges, such as power differentials, limited interdisciplinary collaboration, and inadequate leadership training (12), may differ based on the local healthcare systems and workforce capacity. In South Africa, challenges to effective clinical leadership in labor wards stem from organizational barriers such as hierarchical structures, dynamics within the care environment, low staff capacity to fulfill clinical leadership roles, and the dual burden of clinical and managerial responsibilities (15). These findings correspond with views reported in studies in high-income settings for example clinical leaders lacked leadership training and credentials to assume leadership positions and the structure of their practice was a source of leadership challenges (16), hence presenting difficulty in balancing the needs of the specific patient, client group, or service with the overall needs of the organization and managing tensions and coping with uncertainty and adapting to changing situations (17). Moreover, clinical leaders without proper training may have difficulty navigating the dynamics of a health system, establishing leader–provider–patient relationships, and making informed decisions regarding patient care.

A clinical leadership study found that although clinical leaders may possess clinical leadership qualities for clinical practices, they may not possess the characteristics associated with traditional managerial practice such as being visionary, having control, and being creative (18), and this could highlight a source of disconnect between clinical and managerial skillset that would impact patient care. When clinical leadership challenges are not addressed, they are likely to affect patient care and health system efficiency (15). In Zambia, for example, the negative influences of leadership practices were associated with providers' burnout, demotivation, frustrations, carrying out tasks carelessly, working in fear, and compromised quality of care to patients (19). It could also increase clinical leader stress levels and enhance patient negative outcomes. In a study in a resource-limited setting, clinical leaders were in a dilemma in providing the best patient care due to insufficient resources and perceived higher staff workload and frustration (20). This would also compromise the quality and delivery of patient care through increased wait times, limited access to essential treatment, and lower patient satisfaction (21). A scoping review of the impact of clinical leadership in advanced practice roles outcomes in healthcare revealed a paucity of objective evidence of the impact of advanced practitioners' clinical leadership on patients, staff, or organizational outcomes (22), indicating insufficient evidence of the extent of integrating clinical leadership in advanced practice roles. This would also create a challenge to healthcare institutions on how to leverage clinical leaders' expertise to improve the quality of patient care and may hinder efforts to allocate resources and streamline healthcare processes to enhance health system performance. While substantive literature has utilized quantitative methods to examine clinical leadership in different contexts, a few studies have explored the challenges that clinical leaders encounter in HIV contexts, the sources of the challenges and consequences to HIV care, and the health system performance.

In recognition of the less documented challenges in HIV literature, this study aimed to explore the perspectives of healthcare providers regarding the challenges faced by clinical leaders in the care of people living with HIV/AIDs and considers the sources of clinical leadership challenges and their consequences to HIV care and health system improvement. The study posed the following research question:

1. What are the clinical leadership challenges in HIV care systems as perceived by healthcare providers?

## 2 Materials and methods

### 2.1 Design and setting

We conducted an exploratory qualitative study from December 2019 to May 2020 that involved one-to-one in-depth interviews with healthcare providers working in the Academic Model Providing Access to Healthcare (AMPATH). We selected this approach to explore providers’ perspectives on the challenges of clinical leadership in an HIV care system, and this was shaped by our professional backgrounds and prior experience in healthcare settings. Throughout the study, we remained reflexive about how our assumptions, professional backgrounds, and positionalities could influence data collection, interpretation, and interactions with participants. AMPATH is a consortium of institutions in Kenya including Moi University, College of Health Sciences, Moi Teaching and Referral Hospital, and Universities from Northern America. AMPATH provides comprehensive HIV care to the population living in Western Kenya and provides support to more than 600 Ministry of Health Facilities in 15 counties in Western Kenya. The AMPATH system is organized into five units of care provision referred to as “modules”; Modules 1–3 serve the needs of adult patients, Module 4 serves the children, and Module 5 serves the needs of adolescents (23). The study period coincided with the onset and initial spread of the COVID-19 pandemic, a time when clinical leaders and healthcare providers had increased levels of panic, anxiety, and stress in providing emergency response to address the COVID-19 pandemic alongside managing existing HIV care, hence providing a dual health crisis that shaped the experiences and emotional context of both participants and researchers.

### 2.2 Sampling and recruitment

The study included healthcare providers of different cadres (clinicians, nurses, counselors, social workers, patient retention officers, and pharmacists) who were working in the AMPATH facility. At the time of this study, there were 50 healthcare providers in the facility who worked under the clinical leaders in the facility, and we only sampled those serving in Modules 1–3 for adult patients. Each module was headed by a clinical leader who provided clinical and leadership roles. Before we conducted the in-depth interviews, we sought approval from the AMPATH facility by presenting our research permits. We initially approached 25 healthcare providers using purposive sampling and conducted interviews until we reached data saturation at 22 participants including one pharmacist, 14 clinical officers, five nurses, and two counselors. Purposive sampling is a non-probability method that allows the selection of participants with specific information (24, 25), in this case, to answer our research question on the perceived clinical leadership challenges with the highlighted source, and consequences to HIV care and system improvement. Our purposive criteria included providers with over 1 year of experience in HIV care provision under the supervision of a clinical leader to ensure that the views obtained were directly relevant to the specific clinical and leadership challenges in the HIV setting. We also included providers with diverse professional backgrounds, both male and female of different cadres to ensure the inclusion of diversity in perspectives and participants who were willing to participate in the study voluntarily to ensure the richness and meaningfulness of the collected data. We remained reflexive throughout the recruitment process, particularly regarding our positions and familiarity with the AMPATH setting because these dynamics may have influenced participants' willingness to engage with the study and the openness of their responses. We were also mindful of potential power relations and professional hierarchies within the facility that could affect how participants perceived the purpose of the research or their ability to speak freely. To address this, we emphasized voluntary participation and established rapport to support a safe space for honest dialogue (26).

### 2.3 Data collection

The interviews were conducted by two members of the research team, FC and JW, who had prior experience in community and stakeholder engagement and qualitative research. At the time of the study, FC held an MSc in International Health Research Ethics (Bioethics) and had previously conducted qualitative research in both community settings and among healthcare professionals. JW is a public health expert and has a PhD in Health Behavior and extensive experience in qualitative research. The healthcare providers consented to the in-depth interviews that were done in a room that was identified for the study within the HIV facility to protect privacy and confidentiality and to allow the participants to process information. The authors ensured that the data collection procedures and analysis were transparent and simple to ensure consistency and rigor throughout the study. This was achieved by conducting a pre-interview session to ensure the data collection tool was appropriate to collect accurate data. Throughout this process, we remained conscious of how our identities as researchers, some with familiarity with the health system could influence participants' responses. In line with (26), we emphasized mutual respect and mitigated power dynamics through open dialogue and reassuring the voluntary nature of participation in the study. We developed an interview guide with semi-structured questions that were derived from the research question to obtain clear responses from the participants on the perceived clinical leadership challenges in an HIV care system, highlighting the sources of the clinical leadership challenges, and their consequences to HIV care and health system improvement. We also included participant demographics. The in-depth sessions were audio-recorded on participant consent and took at most 1 h.

### 2.4 Data management and analysis

Data analysis was guided by a thematic approach (25, 27). First, the audio-recorded interviews were transcribed verbatim by FC and then translated and verified by two authors (FC and JW) with qualitative backgrounds as accurate to ensure rigor and consistency in the analysis. We adopted the six steps outlined by Braun and Clarke (55) including familiarization with the data, generating codes, searching for themes, reviewing themes, defining and naming themes, and locating exemplars. First, all the authors (FC, AK, MK, VB, VN, and JW) read the transcripts for familiarization to understand the richness of the data. The transcribed data were then imported to the NVivo software v.12 for initial coding and managing data ideas and queries. Then, two authors (FC and JW) developed the initial codes to organize the data into meaningful categories that were used to identify recurrent and related themes. The codes with coded data were then reviewed collaboratively by all authors to identify broader patterns in the data and grouped similar codes that defined the potential themes. The themes were then refined by all the authors to ensure they were accurate, clear, and distinct, provided valuable insights into the research questions, and represented the data. Any duplicate ones were removed, and a final codebook was developed that informed the final write-up of the report supported by illustrative quotes from the data (28–30). Throughout the research process, we engaged in reflexive (26) team debriefing sessions to continuously examine our assumptions and potential biases and the potential for interpretative subjectivity. In this, all authors reviewed the coded data to minimize individual biases and strengthen the credibility of the findings to maintain analytical rigor. It also helped us remain aware of how our interpretations were shaped by our positionalities and supported transparency in theme development.

## 3 Results

### 3.1 Participant demographic characteristics

There were 22 (88%) participants in the study. Most were clinical officers (14; 63.6%), followed by nurses (5; 22.8%), counselors (2; 9.1%), and a pharmacist (1; 4.5%). Males (12; 54.5%) were slightly more than females (10; 45.4%), earned a monthly income exceeding 500$, and had over 1-year experience providing HIV care with an average age of 41–50 years as shown in Table 1.

**Table 1:** Participant demographic characteristics.

Participant characteristics	*n* (%)
Total participants	22 (88%)
Gender	
Male	12 (54.5%)
Female	10 (45.4%)
Profession	
Clinical officers	14 (63.6%)
Nurses	5 (22.8%)
Counselors	2 (9.1%)
Pharmacist	1 (4.5%)
Monthly income	
(>500$)	22 (88%)
Experience	
>1 year in HIV care	22 (88%)
Age	
(41–50 years)	22 (88%)

### 3.2 Themes and summary of the findings

The study results are organized around three broad themes that were derived from the research questions: perceived challenges of clinical leadership in an HIV care system that are perceived to emerge from the providers (supply side), the patients (demand side), and those from the health system side that are linked to the execution of clinical leader roles and responsibilities in an HIV primary healthcare facility. The findings also highlight the sources of perceived clinical leadership challenges and illuminate the consequences to patient care and health system performance.

### 3.3 Clinical leadership challenges

#### 3.3.1 Supply-side challenges

##### 3.3.1.1 Resources shortage

Participants highlighted the shortage of resources as a key challenge to the clinical leader which included a shortage of staff and a lack of some commodities and supplies. In their view, understaffing led to a high patient ratio against the number of staff in the various departments which would present a dilemma to the clinical leader in scheduling tasks and responsibilities among the few staff across departments and could cause poor planning leading to critical care gaps. This may also create inefficiency in providing HIV care. At the time of this study, AMPATH Plus was facing a budget cut from the donor United States Agency for International Development (USAID) which led to staff layoffs. As a result, it created negative consequences on sustained HIV care for example patient retention in HIV care. In addition, there were times when the staff could be on annual leave, were transferred to other departments, or could have fallen sick, and there was no one to cover for them. This led to long waiting hours in service provision to the patient and unmet objectives of the clinical sections where the leader heads.

Challenges are human resource challenges in terms of personnel, that is why I was telling you at the moment we don’t have support staff who have been helping us in making phone calls to patients. You see we are dealing with HIV clients and some definitions like if a patient misses to come after 28 days, you term/call/define that as a loss to follow-up (LTFU), so you need to follow up cases. So, peers have been helping us in terms of follow-ups. (CO1)

So currently, the challenges we are facing now, we have the issues of budget cuts, staff are being laid off, there are the issues of anxiety so that even those who have not received their termination letters are not stable, are not stable because you are not sure if you are going to be the next. (CO3)

In addition, a shortage of essential supplies and equipment was perceived as a resource challenge that constrained the clinical leader in executing clinical functions. This included stock-outs of drugs, inadequate machines, or even the machines available being out of order and needing some repair, which resulted in dwindling donor funding. In this case, the clinical leader would be required to make tough decisions in allocating scarce resources that will oversee the running of the clinic.

Then there are also the necessary resources, in terms of tools of work that are mostly not adequate. A client may need some services but you are unable to offer them because you do not have the necessary resources. (NR1)

I would say sometimes we don’t have the essential equipment to do our work because maybe because of the budget cut, maybe sometimes we lack funds to go for maybe how will I call, fieldwork or trace patients or maybe assist patients, you know like you can be sick but you have other underlying social issues, eh, economic issues but we cannot come in and she cannot like to support us with us may be budget. (CO2)

A commonly identified challenge was financial constraint which was viewed to impede the clinical leader in discharging daily clinical and leadership functions. Participants noted that a lack of or limited financial adequacy resulted in staff layoffs. This created a high anxiety among staff who were still in the system and were engaged on contractual terms since they were not sure of their job security and stability the leader at the operational level is expected to advocate for them but the leader had no control because it was a high-level decision made by the top leadership.

I think the major challenge is still financing. Because at some point, you might need a few things here and there but it is beyond your control even as a leader in a certain department. (CO4)

For the challenges, for the financial constraint and all that. There are those that we can and those that we can’t handle. You know this is a donor program and there are decisions that a made beyond the facility management level. (NR2)

##### 3.3.1.2 Staff welfare

The participants noted that clinical leaders experienced leadership challenges in managing competing tasks such as high workloads among staff because the HIV clinics were always busy and attending frequent departmental meetings, hence leading to pending assignments for the clinical leaders due to time constraints.

Leadership comes with challenges, it can never lack challenges because maybe there are competing tasks, some of the challenges, you want to finish this, you look at your diary you have a lot to cover within maybe a short time, so competing tasks is the major challenge I can talk about. (CO6)

The workload is just too much, just like the example I had given you. If you come in the morning, you are attended to nicely, but if you come by mid-morning, you find an irritated healthcare practitioner who is tired. (CO5)

It was noted that a lack of motivation for staff in terms of low salary and job security was a challenge due to low pay and high workload that created a challenging work environment for the clinical leaders through balancing the needs of patients and staff and encouraging them to be productive. As a result of demoralized staff, it affected the quality of the care services provided and team dynamics.

And also, the allowances are low, the salaries are low, and you can’t leave because you don't have any other job. So, you just want although we are not well motivated and the workload is so much. So, by the end of the day, you get a demoralized workforce. (CO5)

A lack of proper training in leadership skills, for example, strategic leadership and management and response to emergencies like the COVID-19 pandemic, affected service delivery. Some of the leaders who held management positions without leadership training ended up struggling in discharging leadership and clinical roles, and this affected quality service provision. In addition, the lack of proper training and capacity building of the care providers was noted to affect quality service provision. Furthermore, participants noted a lack of support from the management in their career progression to improve their leadership skills and acquire new expertise in medical practices to remain competent in their field, enhance continuous improvement, lead teams effectively, and provide high-quality patient care.

Another thing is lack of training. You know, as a leader, you need to attend most of the leadership training so that you can equip yourself with knowledge. So sometimes we raise for leadership training but we are not sponsored or supported in a way. (CO7)

When you look at things like emergencies, you will realize that probably you don't have much staff trained in emergencies so people might feel that their patient was not treated well in that hospital compared to how they would have been treated in another hospital. (CO4)

One of the challenges that our leaders face is lack of support. Lack of support from the management above him, especially when it comes to training. Maybe you are told that there are no funds. (CO5)

##### 3.3.1.3 Team dynamics

It was further noted that team dynamics was a challenge to the clinical leader in terms of diversity in the characteristics of the staff. For instance, the age difference between the leader and staff members led to some misunderstandings hence impacting decision-making regarding patient care.

Also, sometimes age is a factor. Some people may be senior to the leader by age and the leader may find it so embarrassing to put them in a corner and fix them and have different results. (P1)

In addition, the clinical leaders experienced disrespect and a lack of cooperation or teamwork from some team members due to the leadership approach of the leader, presenting interpersonal challenges that undermine effective leadership and teamwork within the health system. Other providers delay in submitting their section reports, hence affecting the smooth delivery of the services in the care system and overall decision-making.

A leader is someone who should be reliable to the team, but sometimes the team might take advantage of your goodness or might take advantage of the respect that you have for them and think that is a weakness, which is not really and I am trying to talk to my team and tell them that not because I respect you does not mean that I am not firm enough to expect results. (P1)

Sometimes you get a staff member who is not punctual, doesn't want to perform his or her responsibilities, so you have to make sure that they perform according to their duties and job description and if the worse comes to the worst, then you can report to the supervisor so that they can take up the necessary action. (CO7)

Difficulty in the management of the integrated services was a challenge that required the leader to provide effective coordination and communication between different departments.

We have challenges whereby we need to involve other cadres such as nutrition, social work, being a leader to whom we air our issues as care providers, he usually faces challenges. (CO8)

#### 3.3.2 Demand-side challenges

Participants viewed unmet patient expectations as a challenge to the clinical leader. They noted that some patients had diverse needs that the clinical leader and the providers had no control over. In this case, the leader is required to provide an update about the affected patients and develop appropriate strategies to address the unique needs of the patients, hence facilitating a smooth patient process to receive quality care that meets their expectations.

Different patients have different needs. Sometimes it is difficult to make it universal for all patients and yet the complaints could be from one of two patients. (O5)

Providers noted further that there was a lack of appreciation from the patients for the services they received, and this triggers stress and emotional strain on the clinical leader and the staff because they feel demoralized if their efforts to provide quality care are not appreciated.

Sometimes the patient, he or she cannot appreciate what our staff does. Sometimes there are complaints that they are not getting quality healthcare, they are delayed, they have so many complaints and they direct them to the leader. (NR3)

In addition, patients complained about delays in service provision due to long queues, and this presents a leadership challenge of managing patient dissatisfaction.

People might feel very devastated when they are in line waiting for the services and they might think this hospital is bad and it’s been a long time, you can even die in the queue, but when you look at it keenly, maybe it could be a systemic issue where you don't have enough staff. (CO4)

Participants noted that both the patients and healthcare providers were dynamic in character, and it would pose a challenge to the clinical leader in managing the ever-changing dynamics within an interactive HIV care system which might complicate management within teams and ensure quality patient care provision.

Challenges do not come from one point or side. It could be that the challenges come from patients. We care providers also cause them, for example, pressure from care providers in terms of patient load. (CO8)

Furthermore, participants mentioned that there was a stigma related to being an HIV/AIDs patient. This affected the health-seeking behavior of the patient whereby they would fear and feel ashamed of seeking treatment and medication.

.If we look at the social bit of it, there is stigma for HIV and however much the team could do a lot to support and ensure that the patient gets the best HIV care, number one is they ensure that the patient is identified and tested. But because of stigma, it is not easy for them to come for testing. Having clients who have already been introduced to ART is sometimes hard because of the economic circumstances, stigma, change of location, this being a town and other challenges. (NR2)

#### 3.3.3 Health system-side challenges

The structure of workflow and way of operation in the facility emerged as a challenge to the efficient and quick service provision in the health facility as the clinical leader will strive for efficient and timely service provision to the patient through coordination as asserted by one of the care providers:

By system breakdown I mean, ideally, we are supposed to come in the morning then we see patients, the nurses help to triage the clinicians with the patients, send the patients to the pharmacy, so if the nurse is not there, the system will not work because ideally, the patients should start from the nurse to triage to the clinicians, so sometimes or like the computer, the IT people if we having problems with the computer, the IT people don’t come. So those are some of the challenges that I usually see the leadership going through. (CO10)

Participants noted a lack of appreciation and recognition by the management for the targets being met by clinical leaders in the departments they are heading. As a result, it diminishes work morale and job satisfaction and affects overall work performance.

Then another thing, we feel that the management does not appreciate what we do. It does not appreciate the targets that we meet. It’s just that you meet this target, they bring another target. You meet this, they bring another target, not appreciating what you already did. (CO5)

Participants noted that some services were not available at the facility due to a lack of equipment and specialists. They expressed concerns about a lack of political will at a higher level and poor health system management in providing the necessary resources. This would possibly make clinical leaders work difficult in coordinating the provision of comprehensive HIV care and could lead to service provision gaps that may compromise the quality of patient care.

When you look at the healthcare system in Kenya, let’s not say it is a national problem, but a national healthcare system issue. I may point out that probably the political influence, in a way, has affected the healthcare system because people have different challenges and probably the areas were not well. Let me not say balanced, but you cannot compare the healthcare system in a national referral hospital like MTHR to a county referral hospital. (CO4)

Participants viewed a challenge in communication breakdown when clinical leaders are passing information to the junior staff, due to long processes in the health system that are bureaucratic, causing delays in the procurement process that lead to delays in sharing important information that will inform service provision to the patients. It will also negatively influence teamwork and communication and potentially compromise clinical decision-making and the overall quality of patient care.

But the most glaring challenge is that being below somebody else that you may depend on several issues like procurement, like staff employment, and so forth. Your hands may be tied. You may come on the ground and find that people are overwhelmed and you need staff, but you see that the process of hiring staff is very long and your people are tired on the ground. You may need something to assist you in the service delivery, your people are asking for it, but by the time you get to the procurement, it may even take months and that is also a challenge. It may pull down your efforts while trying to bring out something better. (NR2)

## 4 Discussion

Our study presents findings that describe the challenges facing clinical leaders while providing clinical and leadership functions in the HIV care system from the perspective of healthcare providers. Through semi-structured interviews with 22 healthcare providers, their views were explored using a thematic approach to answer the research questions. Overall, participants perceived clinical leaders to experience challenges that emerge from the supply, demand, and the health system. Putting these factors into context is important in informing leadership interventions that will enhance the effectiveness of HIV care provision in HIV care systems.

### 4.1 Overall challenges

Participants perceived inadequate funding as a challenge that impedes clinical leaders in providing clinical and leadership functions by compromising healthcare quality. Consistent with current literature, health financing has been highlighted as a challenge in improving healthcare operations and patient care (31). The findings further highlighted the stigma related to being an HIV/AIDs patient that makes patients feel socially excluded. This affects patients’ health-seeking behavior, hence making them feel ashamed of seeking treatment and medication. Previous studies reported consistent results that patients experienced stigma and HIV literacy as factors that contributed to a lack of engagement in care (32).

### 4.2 Supply-side challenges

On the supply-side challenges, the study findings highlight the shortage of resources including shortage of staff and lack of commodities and supplies as an impediment to providing quality HIV care to the patient by the provider. This highlights the linkage between resource deficits to broader systemic issues such as strain on healthcare providers that cause burnout and decreased productivity which impact HIV patient outcomes. As a result, it calls for innovative approaches to resource mobilization and management to enhance the overall HIV system effectiveness. The study findings concur with previous studies that cited inadequate resources such as supplies, staff, and drug stock-outs which contribute to poor leadership (33–35). Similarly, there was a shortage of finance, materials, and supplies as a leadership challenge (20, 36). As a result of the patient high volume, providers experienced a high workload suggesting the need for increased capacity to handle the numbers. In the literature, a study suggested having administrators assume leadership positions to lessen the workload on the leader (37). Similarly, the providers perceived their leaders to feel demotivated in their leadership roles due to the low pay that comes with additional responsibility. Ideally, the more the responsibilities, the higher the pay, and this was on the contrary as perceived by the providers. This indicates a perceived disconnect between increased responsibilities and compensation hence impacting the effectiveness of clinical leaders in taking the lead in their responsibilities. A similar study in Nigeria provided a contrary view that involving clinicians in organizational leadership roles was non-beneficial either for patient care or their professional success and therefore it bore irrelevance to the self-esteem and profession of clinicians. Similarly, leadership was not seen as a benchmark for clinical practice (12). In addition, clinical managers were reluctant to take up leadership as a career; hence, they felt some discomfort that could trigger conflict between providing patient care and leadership at the same time (38).

Our findings concurred with previous literature that a lack of capacity building in leadership skills for clinical leaders to deliver their practice was a challenge (16). It is possible to say that when clinical leaders have insufficient training, they are less likely to manage HIV care services and address existing and emerging challenges in the provision of HIV care, for example, managing provider–patient relational dynamics. Consistent with previous literature, clinical leaders in the labor ward, formally appointed clinical leaders, struggled to balance managerial and clinical leadership roles (15). This would be difficult in providing effective clinical practice because the clinical leaders would require diverse leadership attributes and capacities to drive innovative HIV care, hence the need for flexible career growth and development opportunities. Consistently, the leadership and management training in Nigeria was not embedded in the core curricula of clinical leaders, hence the available options for the clinician were external making it difficult to focus on the practical experience of daily challenges (12). A previous study emphasized that communication is not only an asset in clinical leadership roles in coordinating tasks and consultation (39), but it is also considered a key factor in building relationships and delivery of quality medical care in a healthcare setting (40). Contrary to this finding, our study indicated that miscommunication was a challenge to the clinical leader, specifically in passing information to the junior staff, and acknowledged the bureaucratic nature of the facility when procuring materials for service provision. This leads to inefficiency in disseminating important information for decision-making, potentially leading to delays, errors, and even misunderstandings in service delivery (41). Consistent with this finding, a study identified poor communication among patients and nurses where care providers dominate the process hence neglecting patient needs and concerns (42, 43). In the HIV facility, participants perceived that the clinical leader faced challenges related to team dynamics such as staff layoffs and age differences which led to misunderstandings and enmity between the leader and staff. The issues increase stress disrupting the provision of HIV care and require clinical leaders to identify the challenges to understand how the dynamics can be mitigated (15, 44). Similarly, leading and managing teams in an evolving situation is a challenge (45). This was contrary to a previous study where clinical leaders treated team members with respect and facilitated a conducive work environment (46, 47).

### 4.3 Demand-side challenges

Our findings established that unmet patient expectations made some patients become difficult patients because of their diverse needs that the leaders and providers have no control over. The possible interpretation may be attributed to patient–provider relational dynamics and facility management's ability to provide adequate resources that could limit or reduce patient frustrations and dissatisfaction with HIV care. For example, when patients are not satisfied, they tend to get disappointed (21, 48). Moreover, balancing the needs of the patient with those of the organization, as does the degree of focus on the frontline or corporate leadership presents a challenge (17). A study further highlighted that clinicians used available strategies to meet patient expectations by setting goals and using diagrams, fact sheets, and web links for educational and health literacy among patients (36). While clinical leadership is dependent on developing an honest, open, transparent, person-centered relationship between patients, careers, and significant others (8), there is however insufficient literature that discusses patient–provider relational dynamics from a clinical leadership perspective. Our study found that providers perceived some patients to lack a sense of appreciation for the client service they received and they complained about service delays, long queues, and a lack of incentives. It highlights a disconnect between the quality of care and patient expectations. We also believe there is a huge disconnect of transition from a previously organized-funded system that provided incentives with a new care system that does not provide due to funding shortages, creating dissatisfaction and a negative perception of the facility thereby creating poor relationships in the health system.

### 4.4 Health system-side challenges

In our study, providers perceived a rigid organizational structure as a challenge to the clinical leaders' operations particularly when there is no proper coordination of tasks and the appropriate technology to support the operations. The possible argument is that probably the type of leadership and management is a top-down approach and the clinical leaders lack opportunities to exercise full control of their clinical and leadership functions, hence possibly creating inefficiencies in patient service delivery, for instance, clinical leaders may struggle in allocating tasks that are aimed at patient and health system improvement. Furthermore, without management support on all aspects supporting patient care, it makes it difficult for the clinical leader to perform duties, underscoring the importance of a supportive organizational structure and leadership framework to optimize healthcare delivery. Previous studies have cited similar challenges such as complex organizational structures characterized by centralization and bureaucracy and other contextual factors such as regulations and programs (49). In Uganda, verticalized HIV programming has been sustained by rising HIV client loads and external HIV funding systems (50). Our findings further revealed a challenge in the management of integrated services particularly when the patient requires multiple attention for different cases in various departments due to the complex nature of the health system that requires collaboration and effective communication among leaders to meet the diverse needs of the patients. In addition, some essential services in the facility were unavailable due to a lack of equipment and specialists, highlighting a gap in the healthcare infrastructure. Previous studies acknowledge health system factors influence patient care (51, 52). Similarly, a qualitative study found that health systems hardware and software factors such as inadequate infrastructure to protect privacy, delayed opening time, and inflexibility in visit schedules, influenced patient disengagement among lost to follow-up (LTFU) patients (53). Furthermore, health system factors constrain providers in providing quality HIV care (54).

### 4.5 Implications for practice

Our study findings provide evidence for the need for health system leaders to address the existing clinical leadership challenges that improve clinical practice and the provision of HIV care in HIV primary healthcare systems.

### 4.6 Strengths and limitations

The strength of this study was the rich qualitative views from the providers who have been interacting with their leaders. They were able to describe the challenges their leadership experienced, hence increasing confidence in the findings. Moreover, the in-depth views provide a clear picture to the leadership and management of the challenges that the clinical leaders face and a chance to address them. Our findings also are similar to previous studies concerning challenges facing clinical leaders; the difference is that we interviewed them on leadership challenges facing clinicians in an HIV care system.

The limitations of this study include the narrow lens on comparing the results with other clinical leadership challenges, but we specified that these involved views of the providers. In addition, we only obtained the views of the healthcare providers in an HIV setting, limiting the views of the clinical leaders in leadership positions who could have provided different views and experiences on leadership challenges. This study was conducted during the COVID-19 pandemic, and there is a possibility that the providers experienced emotional and psychological distress which could have influenced their responses hence potentially leading to biases and underreporting of certain clinical leadership challenges. Moreover, due to increased workload, providers' priorities may have changed affecting their availability and willingness to participate in the study. There were difficulties in accessing the participants in the facility due to restricted access and safety concerns which delayed interviews and affected the diversity of participants. Furthermore, our interpretation of the findings could have been influenced by our disciplinary and cultural backgrounds. We acknowledge that our focus on participant perspectives may stem from our continuous engagement in participatory research. To address this, all the authors critically engaged in different perspectives during data analysis to present a balanced understanding. These reflexive views enhanced the transparency and trustworthiness of our study and aligned with best practices in the qualitative research of Olmos-Vega et al. (26).

## 5 Conclusion

The results from the study illustrate that in HIV care systems, clinical leaders are faced with diverse challenges that emerge from the supply, demand, and health system sides that affect patient care and system performance. These include resource shortages, unmet patient expectations, and system inefficiencies such as lack of management support that create operational challenges and strain clinical leaders' capacity to provide quality HIV care. This provides a forward-thinking approach to the healthcare facility leadership and management to address these challenges with innovative strategies that include strengthening management support systems and leadership training for clinical leaders to improve HIV care provision. Similarly, clinical leaders should be given opportunities to participate in strategic leadership decisions based on their hands-on experience in leadership at the micro level.

## Data Availability

The original contributions presented in the study are included in the article/Supplementary Material; further inquiries can be directed to the corresponding author.

## References

[1] Budak F Özer Ö. (2018). Exploring the impacts of personal factors on clinical leadership in a university hospital. J Res Nurs, 23(8), 711–24. 10.1177/1744987118788716 34394493 PMC7932419

[2] Daly J Jackson D Mannix J Davidson P Hutchinson M. The importance of clinical leadership in the hospital setting. J Healthc Leadersh. (2014) 6:75. 10.2147/JHL.S46161

[3] Mrayyan MT Algunmeeyn A Abunab HY Kutah OA Alfayoumi I Khait AA. Attributes, skills and actions of clinical leadership in nursing as reported by hospital nurses: a cross-sectional study. BMJ Leader. (2023) 7:203–11. 10.1136/leader-2022-000672 PMC12038119 37192110

[4] Nzinga J McGivern G English M. Examining clinical leadership in Kenyan public hospitals through the distributed leadership lens. Health Policy Plan. (2018) 33(Suppl 2):ii27–34. 10.1093/heapol/czx167 30053035 PMC6037084

[5] Jonas S McCay L Keogh SB. The importance of clinical leadership. In: Swanwick T, McKimm J, editors. ABC of Clinical Leadership, 1st ed. Oxford: Blackwell Publishing Ltd. (2011). p. 1–3.

[6] Mianda S Voce AS. Conceptualizations of clinical leadership: a review of the literature. J Healthc Leadersh. (2017) 9:79–87. 10.2147/JHL.S143639 29355250 PMC5774456

[7] Cherop F Wachira J Bagire V Korir M. Leading from the bottom: the clinical leaders roles in an HIV primary care facility in Eldoret, Kenya. PLoS One. (2024) 19(5 May):1–14. 10.1371/journal.pone.0302066 PMC11142606 38820443

[8] McSherry R Pearce P. What are the effective ways to translate clinical leadership into healthcare quality improvement? J Healthc Leadersh. (2016) 8(February 4):11–7. 10.2147/jhl.s46170 29355204 PMC5741002

[9] Næss G Wyller TB Gjevjon ER. Clinical leadership—an important precondition for the success of proactive and interdisciplinary follow-up of frail older recipients of home healthcare. Home Health Care Manag Pract. (2023) 35(4):242–52. 10.1177/10848223231170599

[10] Mwangi LW Macharia W Wachira BW Kimeu J Mativa B Atwoli L. Role of hospital leadership in pandemic preparedness: experience at a tertiary hospital in Kenya during the COVID-19 pandemic. BMJ Leader. (2023) 8:111–8. 10.1136/leader-2023-000833 PMC12038139 37567757

[11] Nhongo D Holt A Bail K Flenady T. Registered nurses’ confidence related to undertaking a leadership role in residential aged care: a clinical leadership self-assessment survey. Collegian. (2024) 31:202–10. 10.1016/j.colegn.2024.04.004

[12] Donald DU. Challenges of clinical leadership in Nigeria challenges of clinical leadership in Nigeria. J Psychiatry. (2015) 18(1):1–4. 10.4172/2378-5756

[13] Agbodzakey JK. Leadership in collaborative governance: the case of HIV/AIDS health services planning council in South Florida. Int J Public Adm. (2021) 44(13):1051–64. 10.1080/01900692.2020.1759627

[14] Algunmeeyn A Mrayyan MT Suliman WA Abunab HY Al-Rjoub S. Effective clinical nursing leadership in hospitals: barriers from the perspectives of nurse managers. BMJ Leader. (2023) 8(1):20–4. 10.1136/leader-2022-000681 PMC12038153 37248037

[15] Mianda S Voce AS. Enablers and barriers to clinical leadership in the labour ward of district hospitals in KwaZulu-natal, South Africa. BMJ Leader. (2019) 3(3):75–80. 10.1136/leader-2018-000130 PMC6267734 30568524

[16] Spehar I Sjøvik H Karevold KI Rosvold EO Frich JC. General practitioners’ views on leadership roles and challenges in primary health care: a qualitative study. Scand J Prim Health Care. (2017) 35(1):105–10. 10.1080/02813432.2017.1288819 28277051 PMC5361414

[17] Edmonstone J. Six challenges facing clinical leadership. Br J Health Care Manag. (2020) 26(8):1–6. 10.12968/bjhc.2019.0108

[18] Stanley D Stanley K. Clinical leadership and nursing explored: a literature search. J Clin Nurs. (2018) 27(9–10):1730–43. 10.1111/jocn.14145 29076264

[19] Mulenga RM Nzala S Mutale W. Establishing common leadership practices and their influence on providers and service delivery in selected hospitals in Lusaka province, Zambia. J Public Health Africa. (2018) 9(3):a948. 10.4081/jphia.2018.823 PMC6379688 30854175

[20] Busari JO Yaldiz H Gans ROB Duits AJ. Clinical leadership as an agent for change: a health system improvement intervention in Curaçao. J Multidiscip Healthc. (2020) 13:787–98. 10.2147/JMDH.S262415 32884278 PMC7431449

[21] Wung BA Peter NF Atashili J. Clients’ satisfaction with HIV treatment services in Bamenda, Cameroon: a cross-sectional study. BMC Health Serv Res. (2016) 16(1):280. 10.1186/s12913-016-1512-5 27431998 PMC4950718

[22] Duignan M Drennan J McCarthy VJC. Impact of clinical leadership in advanced practice roles on outcomes in health care: a scoping review. J Nurs Manag. (2021) 29(4):613–22. 10.1111/JONM.13189 33098329

[23] AMPATH. History—AMPATH Kenya (n.d.). Available at: https://www.ampathkenya.org/history (Accessed June 8, 2022).

[24] Reiter B. Theory and methodology of exploratory social science research theory and methodology of exploratory social science. Int J Sci Res Methodol. (2017) 5(4):130–50. Available at: https://hdl.handle.net/2346/86610

[25] Schreier M. Qualitative Content Analysis in Practice. London: SAGE Publications (2012).

[26] Olmos-Vega FM Stalmeijer RE Varpio L Kahlke R. A practical guide to reflexivity in qualitative research: AMEE guide No. 149. Med Teach. (2023) 45(3):241–51. 10.1080/0142159X.2022.2057287 35389310

[27] Schreier M. Qualitative content analysis. In: Flick U, editor. The SAGE Handbook of Qualitative Data Analysis. Los Angeles, CA: SAGE Publications, Inc. (2014). p. 170–83. 10.4135/9781446282243.N12

[28] Braun V Clarke V. Using thematic analysis in psychology. Qual Res Psychol. (2006) 3(2):77–101. 10.1191/1478088706qp063oa

[29] Franklin CS Cody PA Ballan M. Reliability and validity in qualitative research. In: Thyer BA, editor. The Handbook of Social Work Research Methods. Thousand Oaks, CA: SAGE Publications, Inc. (2019). p. 355–74. 10.4135/9781544364902.N19

[30] Kolawole GO Gilbert HN Dadem NY Genberg BL Agaba PA Okonkwo P Patient experiences of decentralized HIV treatment and care in Plateau State, North Central Nigeria: a qualitative study. AIDS Res Treat. (2017) 2017:2838059. 10.1155/2017/2838059 28331636 PMC5346378

[31] Chelagat T Kokwaro G Rice J Onyango J. Addressing health system’s leadership challenges through different problem-solving approaches Strathmore University Business School. J Manag Policy Pract. (2019) 20(2):23–34.

[32] Bofill LM Lopez M Dorigo A Alejandra Bordato ML Cabanillas Fernandez GOS Cahn P Patient-provider perceptions on engagement in HIV care in Argentina. AIDS Care. (2014) 26(5):602–7. 10.1080/09540121.2013.844767 24138788 PMC3966648

[33] Carney M. Public health nurses perception of clinical leadership in Ireland: narrative descriptions. J Nurs Manag. (2009) 17(4):435–45. 10.1111/j.1365-2834.2009.01015.x 19531143

[34] Govender S Proches CNG Kader A. Examining leadership as a strategy to enhance health care service delivery in regional hospitals in South Africa. J Multidiscip Healthc. (2018) 11:157–66. 10.2147/JMDH.S151534 29535529 PMC5836703

[35] Rn DS Rn KS. Clinical leadership and rural and remote practice: a qualitative study. J Nurs Manag. (2019) 27:1314–24. 10.1111/jonm.12813 31162890

[36] Slade SC Hay-smith J Mastwyk S Morris ME Frawley H. Attributes of physiotherapy continence clinicians: a qualitative perspective. Physiotherapy. (2020) 106(2020):119–27. 10.1016/j.physio.2019.01.018 30952466

[37] Jooste K Frantz J Waggie F. Challenges of academic healthcare leaders in a higher education context in South Africa. Educ Manag Adm Leadersh. (2017) 46(4):1–17. 10.1177/1741143216688468

[38] Aufegger L Alabi M Darzi A Bicknell C. Sharing leadership: current attitudes, barriers and needs of clinical and non- ­ clinical managers in UK’s integrated care system. BMJ Leader. (2020) 4:128–34. 10.1136/leader-2020-000228

[39] Galik E. The role of clinical leaders in workforce development and retention in post-acute and long-term care. Caring for the Ages. (2022) 23(4):2. 10.1016/j.carage.2022.04.011

[40] Hudelson P. Improving patient-provider communication: insights from interpreters. Fam Pract. (2005) 22(3):311–6. 10.1093/fampra/cmi015 15805131

[41] Flickinger TE Saha S Moore RD Beach MC. Higher quality communication and relationships are associated with improved patient engagement in HIV care. J Acquir Immune Defic Syndr. (2013) 63(3):362–6. 10.1097/QAI.0b013e318295b86a 23591637 PMC3752691

[42] Beach MC Roter DL Saha S Moore RD Wilson IB. Impact of a brief patient and provider intervention to improve the quality of communication about medication adherence among HIV patients. Patient Educ Couns. (2015) 98(9):1078–83. 10.1016/j.pec.2015.05.011 26021185 PMC4546873

[43] Kwame A Petrucka PM. Communication in nurse-patient interaction in healthcare settings in sub-Saharan Africa: a scoping review. Int J Africa Nurs Sci. (2020) 12(December 2019):100198. 10.1016/j.ijans.2020.100198

[44] Lee E Daugherty JA Hamelin T. Reimagine health care leadership, challenges and opportunities in the 21st century. J Perianesth Nurs. (2019) 34(1):27–38. 10.1016/j.jopan.2017.11.007 29908881

[45] Reay T Golden-Biddle K Germann K. Challenges and leadership strategies for managers of nurse practitioners. J Nurs Manag. (2003) 11(6):396–403. 10.1046/j.1365-2834.2003.00412.x 14641721

[46] Conbere J Heorhiadi A. The challenges of leading healthcare organizations. Theory Pract Socio-Econ Manage. (2018) 3(1):1–13.

[47] Swani J Isherwood P. The approachable team leader: front line perspectives on leadership in critical care. J Patient Saf Risk Manag. (2019) 0(0):1–6. 10.1177/2516043519887045

[48] Naburi H Mujinja P Kilewo C Bärnighausen T Orsini N Manji K Predictors of patient dissatisfaction with services for prevention of mother-to-child transmission of HIV in Dar es Salaam, Tanzania. PLoS One. (2016) 11(10):1–15. 10.1371/journal.pone.0165121 PMC5074583 27768731

[49] Ghiasipour M Mosadeghrad AM Arab M Jaafaripooyan E. Leadership challenges in health care organizations: the case of Iranian hospitals. Med J Islam Repub Iran. (2017) 31(96):108. 10.14196/mjiri.31.96 29951397 PMC6014751

[50] Zakumumpa H Rujumba J Kwiringira J Kiplagat J Namulema E Muganzi A. Understanding the persistence of vertical (stand-alone) HIV clinics in the health system in Uganda: a qualitative synthesis of patient and provider perspectives. BMC Health Serv Res. (2018) 18(1):1–13. 10.1186/s12913-018-3500-4 30185191 PMC6126041

[51] Camlin CS Ssemmondo E Chamie G El Ayadi AM Kwarisiima D Sang N Men “missing” from population-based HIV testing: insights from qualitative research. AIDS Care. (2016) 28(Suppl 3):67–73. 10.1080/09540121.2016.1164806 27421053 PMC5749410

[52] O’Brien N Hong QN Law S Massoud S Carter A Kaida A Health system features that enhance access to comprehensive primary care for women living with HIV in high-income settings: a systematic mixed studies review. AIDS Patient Care STDS. (2018) 32(4):129–48. 10.1089/apc.2017.0305 29630850

[53] Mwamba C Sharma A Mukamba N Beres L Geng E Holmes CB They care rudely!’: resourcing and relational health system factors that influence retention in care for people living with HIV in Zambia. BMJ Glob Health. (2018) 3(5):1–11. 10.1136/bmjgh-2018-001007 PMC6231098 30483408

[54] Genberg B Wachira J Kafu C Wilson I Koech B Kamene R Health system factors constrain HIV care providers in delivering high-quality care: perceptions from a qualitative study of providers in Western Kenya. SAGE. (2019) 18:1–10. 10.1177/2325958218823285 PMC6503317 30798666

[55] Braun V Clarke V. Thematic Analysis: A Practical Guide. Los Angeles, CA: Sage Publications (2021). p. 1–100.

