# Supplementary material for: A qualitative study on the challenges of clinical leadership in an HIV care system: insights from healthcare providers in Eldoret, Kenya
Source: Front Health Serv. 2025 May 14;5:1404902. doi: 10.3389/frhs.2025.1404902 (PMC12116568; doi:10.3389/frhs.2025.1404902)
Supplement: Supplementary file 1 [file Datasheet1.pdf]

Supplementary material_S1: Interviewer Guide

Focus Area/Domains	Examples of questions and probes
Study Participation and Introduction	Welcome<br>Description of the study and interview process
Healthcare system leadership challenges	• At the moment, what do you think are the challenges related to clinical leadership in this HIV health system<br>• Where do these challenges emerge from/source?<br>• What are the possible consequences to HIV patient care and the healthcare system's performance?
Thank you	Thank you for your participation in this study

Supplementary material_S2: Codebook with minimal data

3, Challenges	Clinical leadership challenges	21	154
1. Leadership and health care system	The challenges related to clinical leadership and the healthcare system	21	90
Age difference with the juniors	"Also, sometimes age is a factor. Some people may be senior to the leader by age and the leader may find it so embarrassing to put them in a corner and fix them and have different results" (Pharmacist)	1	1
Budget cuts	"So currently, the challenges we are facing now, we have the issues of **budget cuts,** staff are being laid off, there are the issues of anxiety so that even those who have not received their termination letters are not stable, are not stable because you are not sure if you are going to be the next" (Clinical officer)<br><br>"And then another issue is the issue of the contract. You know you have a contract. You have been on a perennial contract. Every year another contract, like this January we don't have a contract so you are working and you don't know" (Clinical officer)	1	1
Competing tasks	"Leadership comes with challenges, it can never lack challenges because maybe there are competing tasks, some of the challenges, you want to finish this, you look at your diary you have a lot to cover within maybe a short time, so competing tasks is the major challenge I can talk about" (Clinical officer)	1	1
Difficulty in managing integrated care	"We have challenges whereby we need to involve other cadres such as nutrition, social work, being a leader to whom we air our issues as care providers, he usually faces challenges" (Clinical officer)	1	2

	Difficult patients to deal with	“Sometimes the patient, he or she cannot appreciate what our staff does. Sometimes there are complaints that they are not getting quality healthcare, they are delayed, they have so many complaints and they direct them to the leader” (Nurse)<br><br>“With the patients, some of them are not even satisfied, despite whatever good thing you provide for them especially here at AMPATH. Initially, we used to give so many things to our patients, including transport, including farm inputs, but nowadays we don't support them. So, patients when they come to learn of it, they think that you are keeping their money or there is a lot of mismanagement. Sometimes they can harass you and tell you that you are eating our money, you are useless, and you don't give us good services... And you are left wondering” (Clinical officer)<br><br>“People might feel very devastated when they are in line waiting for the services and they might think this hospital is bad and it’s been a long time, you can even die in the queue, but when you look at it keenly, maybe it could be a systemic issue where you don't have enough staff” (Clinical officer)	3	3
	Financial challenges	“I think the major challenge is still financing. Because at some point, you might need a few things here and there but it is beyond your control even as a leader in a certain department” (Clinical officer)	3	3
	Financial constraints among patients	“We are a high-volume facility and patients come with different needs, and sometimes you may not be able to assist them because of financial constraints among patients. And then also, for example, laboratory making a diagnosis, you need to send the patient to the lab and they don’t have…you see a patient has a, assume, the patient has a medical cover like they are being covered by NHIF and they don’t have any other source. So, if the facility cannot finance, it is a challenge… Some don’t have even food, they don’t have, and they have to take drugs daily, and you see you cannot provide them with food. Some challenges are beyond us” (Clinical officer)<br><br>“Sometimes patients come and they demand some services and I have no cash and this is a person in need. Leaders now wonder because right now we don’t have a kitty for that and maybe the patient has struggled from Bungoma and has no transport back. The leader will therefore hustle on what to do... On the client has been prescribed for some drugs and he does not have money” (Nurse)	1	2

	High workload	"The workload is just too much, just like the example I had given you. If you come in the morning, you are attended to nicely, but if you come by mid-morning, you find an irritated healthcare practitioner who is tired" (Clinical officer)	3	4
	Inadequate resources and facilities	"Challenges are human resource challenges in terms of personnel, that is why I was telling you at the moment we don't have support staff who have been helping us in making phone calls to patients. You see we are dealing with HIV clients and some definitions like if a patient misses to come after 28 days, you term/call/define that as a loss to follow up (LTFU), so you need to follow up cases. So, peers have been helping us in terms of follow-ups" (Clinical officer)	3	4
	Integrated service provision	"We have challenges whereby we need to involve other cadres such as nutrition, social work, being a leader to whom we air our issues as care providers, he usually faces challenges" (Clinical officer)	1	1
	Lack of appreciation from the management	"Then another thing, we feel that the management does not appreciate what we do. It does not appreciate the targets that we meet. It's just that you meet this target, they bring another target. You meet this, they bring another target, not appreciating what you already did" (Clinical officer)	1	1
	Lack of motivation by staff	"And also, the allowances are low, the salaries are low, and you can't leave because you don't have any other job. So, you just want although we are not well motivated and the workload is so much. So, by the end of the day, you get a demoralized workforce" (Clinical officer)	2	3
	Lack of proper training	"Another thing is lack of training. You know, as a leader, you need to attend most of the leadership training so that you can equip yourself with knowledge. So sometimes we raise for leadership training but we are not sponsored or supported in a way" (Clinical officer)<br><br>"When you look at things like emergencies, you will realize that probably you don't have much staff trained in emergencies so people might feel that their patient was not treated well in that hospital compared to how they would have been treated in another hospital" (Clinical officer)	1	1
	Lack of some commodities and supplies	"I would say sometimes we don't have the essential equipment to do our work because maybe because of the budget cut, maybe sometimes we lack funds to go for maybe how will I call, fieldwork or trace patients or maybe assist patients, you know like you can be sick but	3	4

	you have other underlying social issues, eh, economic issues but we cannot come in and she cannot like to support us with us may be budget" (Clinical officer)<br><br>"Then there are also the necessary resources, in terms of tools of work that are mostly not adequate. A client may need some services but you are unable to offer them because you do not have the necessary resources" (Nurse)		
Lack of support from the management	"One of the challenges that our leaders face is lack of support. Lack of support from the management above him, especially when it comes to training. Maybe you are told that there are no funds" (Clinical officer)	2	2
Lack of teamwork from staff	"A leader is someone who should be reliable to the team, but sometimes the team might take advantage of your goodness or might take advantage of the respect that you have for them and think that is a weakness, which is not really and I am trying to talk to my team and tell them that not because I respect you does not mean that I am not firm enough to expect results (Pharmacist)<br><br>"Sometimes you get a staff member who is not punctual, doesn't want to perform his or her responsibilities, so you have to make sure that they perform according to their duties and job description and if the worse comes to the worst, then you can report to the supervisor so that they can take up the necessary action" (Clinical officer)	1	1
Miscommunication	"But the most glaring challenge is that being below somebody else that you may depend on several issues like procurement, like staff employment, and so forth. Your hands may be tied. You may come on the ground and find that people are overwhelmed and you need staff, but you see that the process of hiring staff is very long and your people are tired on the ground. You may need something to assist you in the service delivery, your people are asking for it, but by the time you get to the procurement, it may even take months and that is also a challenge. It may pull down your efforts while trying to bring out something better" (Nurse)	1	2
Stigma on HIV and AIDs	"If we look at the social bit of it, there is stigma for HIV and however much the team could do a lot to support and ensure that the patient gets the best HIV care, number one is they ensure that the patient is identified and tested. But because of stigma, it is not easy for them to come for testing. Having clients who have already been	1	1

	introduced to art is sometimes hard because of the economic circumstances, stigma, change of location, this being a town, and other challenges" (Nurse)		
Unavailable services in the facilities	"When you look at the healthcare system in Kenya, let's not say it is a national problem, but a national healthcare system issue. I may point out that probably the political influence, in a way, has affected the healthcare system because people have different challenges and probably the areas were not well. Let me not say balanced, but you cannot compare the healthcare system in a national referral hospital like MTHR to a county referral hospital" (Clinical officer)	1	1
Unmet demands by staff		1	1
Unmet patients expectations	"Different patients have different needs. Sometimes it is difficult to make it universal for all patients and yet the complaints could be from one of two patients" (Clinical officer)	4	4
2. Emerge from	Where challenges emerge from	16	29
Dependence on donor funds	"And then another thing, being an NGO, we cannot blame also because sometimes the NGO cuts the funding as like now, we have fund-cut to Kenya. So, they reduce funding and they expect us to perform. They are laying off staff yet they want us to meet the target" (Clinical officer)<br>"Because if you have money, you will have enough supply. If you have money, you will have enough staff. You will provide your patients with whatever they need. Transport; we used to have, but not now. We used to have all these some years back. We used to have money for transport and drugs, but not anymore" (Nurse)	1	1
Patients	"Challenges do not come from one point or side. It could be that the challenges come from patients. We care providers also cause them, for example, pressure from care providers in terms of patient load" (Clinical officer)	3	3
Poor leadership	"The challenges we can say that maybe from the leadership itself, because if, they are the deciding factors, if they have decided that we are doing this, then we cannot say no because who are we to say no? if this thing is not happening and they have said, and also they know this person is not there, so we depend on them but what you say, is what we will follow" (Clinical officer)<br>"One thing I would like to say is that we have a leadership that does not know what goes on in patient care. They don't know. So, sometimes decisions come from up not taking into	3	3

	consideration the clinician who maybe sees sixty patients in a day goes through" (Clinical officer)		
Rigid systems of operations	"By system breakdown I mean, ideally, we are supposed to come in the morning then we see patients, the nurses help to triage the clinicians with the patients, send the patients to the pharmacy, so if the nurse is not there, the system will not work because ideally, the patients should start from the nurse to triage to the clinicians, so sometimes or like the computer, the IT people if we having problems with the computer, the IT people don't come. So those are some of the challenges that I usually see the leadership going through" (Clinical officer)	1	1
Work environment	"Generally, in a work environment, people are different. It comes on any normal day and issues will come up. Someone might not be at their workstation. A patient needs to be seen and the person who is supposed to attend to them is not there and they didn't tell another person to cover up for them for that particular time" (Pharmacist)	2	2
3. Consequences	The possible consequences to patient care and the entire healthcare system	19	35
Frustrations from the healthcare providers	"Because if you are behind the information, for example, if there are new guidelines and you have not been shown a way on how to implement them, you cannot deliver the right service to the client using the updated guidelines" (Clinical officer)	1	1
Overworked staff	And then the workload. The workload is just too much, just like the example I had given you. If you come in the morning, you are attended to nicely, but if you come by mid-morning, you find an irritated healthcare practitioner who is tired" (Clinical officer)	1	1
Poor attitude and relation between the staff and the patient	"If someone is too tired again the service delivery or service provision for the patient won't be the same as someone who is not eh, so usually you will find that at some point or the other, the attitude from the providers to the patient change, and the attitudes of the patient towards provider will not be the same because you will find that if someone is too tired, the rate at which services being given to the patient, will take quite long as compared when somebody is not tired. So, you will find that waiting time for a patient will be quite long as compared to other scenarios" (Clinical officer)	2	2
Poor quality of services to the patients	"It is us (providers) who impact patient care because they are not getting the quality services that they are supposed to be offered to them. Because of that lack of staff, they meet sub-standard service which compromises the quality of the services" (NR3)<br>"The patients are mostly disappointed, and	9	9

	especially the ones who are poor and cannot afford because we end up losing patients because all those things have not been considered" (Clinical officer)		
